# Nutritional habits among nursing students using Moore Index for Nutrition Self Care: A cross‐sectional study from the nursing school Riyadh, Saudi Arabia

**DOI:** 10.1002/nop2.572

**Published:** 2020-07-26

**Authors:** Adel Bashatah

**Affiliations:** ^1^ Department of Administration and Education College of Nursing King Saud University Riyadh Saudi Arabia

**Keywords:** diet, habits, nurses, nursing students, Saudi Arabia

## Abstract

**Background:**

The topics of under‐nutrition and over‐nutrition are important and have significant roles in adolescent life. These concepts must be explored in adolescents owing to the undesirable effects that being underweight or overweight causes.

**Purpose:**

To evaluate the nutrition habits among adolescents from King Saud University nursing school in Riyadh, Saudi Arabia using the Arabic Version of Moore Index for Nutrition Self Care.

**Method:**

This was a cross‐sectional study carried out at a King Saud University nursing school campus to evaluate the Nutrition habits among adolescents. Self‐care practises related to nutrition were assessed by using a self‐report questionnaire that consisted of 50 items assessed on a five‐point Likert scale. Data were collected between January to March 2019.

**Findings:**

The study results revealed that most students 120 (85.7%) had fair nutritional habits, while 11 (7.9%) had good nutritional habits and 9 (7.9%) had poor nutritional habits. There was a significant difference between those who exercised regularly (mean 53.42) and those who did not exercise regularly (mean 46.83; *p* = .002). In addition, simple linear regression revealed that working out regularly was associated with the score on the nutritional questionnaire (*β* = −6.41; *SE* 2.5, *p* = .01). Results revealed no significant associations between gender or age and (*p *= .8; *p *= .9) nutritional score.

**Conclusion:**

To increase levels of good nutritional habits and awareness, it may be useful to adopt educational programs for dietary consumption and physical training promotion.

## 1 INTRODUCTION

In this modern era, under‐nutrition and over‐nutrition are important topics to consider. These concepts must be explored in adolescents owing to the undesirable effects that being underweight or overweight cause (Čakar, Šobajić, Vidović, & Đorđević, 2018). Adolescence is an age characterized by rapid physiological changes including growth; it is during adolescence that secondary sex characteristics develop, which requires more energy and healthy nutrition (Martins et al., 2011). The dietary habits of the adolescents (e.g. consumption of meals, preference for certain meals and selection of meals) and social activities of adolescents are greatly influenced by the socio‐economic conditions of the family (Hagman, Bruce, Persson, Samuelson, & Sjölin, 1986; Samuelson, 2000). Research has indicated that poor nutritional habits result in chronic disease such as diabetes (Afshin et al., 2015; Memish et al., 2014; Moradi‐Lakeh et al., 2017).

Previous studies in Saudi Arabia (SA) revealed the presence of poor dietary habits among school children (e.g. skipping breakfast; a low intake of proteins, fruits and vegetables; a high intake of carbonated beverages, sweets and fast food) (Bin, Musaiger & D'Souza, 2009; Qotba & Naser Al‐Isa, 2007). These poor dietary habits can significantly contribute to the likelihood of being obese or overweight (Farsi & Elkhodary, 2017; Shaikh et al., 2016). The purported cause of these poor dietary habits is the adoption of a more Westernized diet in SA (Gasbarrini & Piscaglia, 2005). In SA, Moradi‐Lakeh et al. conducted a national survey among adolescents in 2016 and they found that adolescents followed poor dietary practises with regard to the consumption of low levels fruits and vegetables, high levels of high‐fat dairy (Moradi‐Lakeh et al., 2017). These researchers also found that the consumption of processed foods and sugar‐sweetened beverages was high among adolescents (Moradi‐Lakeh et al., 2017). Similarly, another recent study by Khayri et al. in SA revealed that 8.75% of the adolescents were either underweight or overweight (Khayri, Muneer, Ahmed, Osman, & Babiker, 2016). A study conducted in 2010 reported a moderate prevalence of underweight adolescents at 6.9% (Mouzan et al., 2010), while a study conducted by Alshammari, Suneetha, Adnan, Khan, and Alazzeh (2017) reported a 4.73% prevalence of being underweight and the prevalence of being overweight or obesity was 20.8%. Several previous studies from SA also established the presence of what is termed double burden malnutrition; this refers to the presence of both under‐ and over‐nutrition among adolescents (Mouzan et al., 2010). However, the results of previous studies revealed that under‐nutrition in SA is limited owing to its economic development and resources (Mouzan et al., 2010).

Practising healthy lifestyle behaviours are challenging, particularly for adolescents (Adel & Khalid unpublished). Past research has indicated that adolescents are prone to poor habits including skipping meals and eating unhealthy foods during leisure time (Čakar et al., 2018; Mouzan et al., 2010). These unhealthy eating habits may lead to several chronic diseases such as obesity (Huang et al., 2003). Given the problems with dietary practises among adolescents, more attention and care are needed since diet can affect both physical and mental development (Nandy, Irving, Gordon, Subramanian, & Smith, 2005). In Saudi Arabia literature suggested that, there is a high incidence of diseases such as high blood pressure and diabetes associated with poor dietary habits (Farghaly, Ghazali, Al‐Wabel, Sadek, & Abbag, 2007). Therefore, the objective of the current study was to assess the nutritional habits among nursing students at a University in Riyadh, Saudi Arabia.

## 2 PARTICIPANTS AND METHODS

### 2.1 Study design, setting and sample

This study used a cross‐sectional design and was conducted at College of nursing king Saud University in Riyadh, Saudi Arabia. Paper‐based questionnaires were administered over a period of 2 months, from January 2019–March 2019. Study included was adolescent males and females between the ages of 18–9 years who were willing to participate and who could understand the Arabic language. Students with speech or hearing disabilities were excluded from the study.

### 2.2 Ethical consideration

The study was approved by the ethics Committee at the college of medicine, King Saud University, Riyadh Saudi Arabia with following reference number (E‐19‐3979). A written informed consent was obtained from the students before data collection.

### 2.3 Measurements/Instruments

To explore the dietary habits among students from King Saud University nursing School, a questionnaire was developed through a review of the literature and with the assistance of members of the research team who had experience in questionnaire development. The survey questionnaire comprised three themes and consists of a total of 50 items (Appendix S1). The first theme was named estimative operations, and it was defined as activities that involve gathering information, acquiring knowledge and identifying alternatives and it consisted of total of 15 items. The second theme was transitional operations, defined as behaviours such as considering various options, making decisions and planning what action needs to be taken with 12 items. The last theme was production operations, defined as identifying resources and evaluating the results of the action to meet the need for self‐care with 17 items all the items were assessed on five point Likert scale (never = 1; rarely = 2; sometimes = 3; most of the times = 4; always = 5). We defined a score of 60 was considered fair nutritional habits, while a score of 50 were considered good and a score of less than 50 were considered poor nutritional habits among the students. We used already translated and validated questionnaire into Arabic, which gave a higher value of Cronbach's alpha of 0.91. We got the necessary permission to use the translated questionnaire from the author (Bashatah & Alahmary, 2020).

### 2.4 Data analysis

Data were anonymized (i.e. personal information like names and addresses were removed). A unique identifier was assigned for data analysis. The data were analysed using SPSS version 22 for Windows. Descriptive statistics included percentages and frequency distributions. The bivariate *t* test and chi‐square test analysis were carried out to find the association between the variables. The level of statistical significance was set at *p* ≤ .05.

## 3 RESULTS

In total, 140 students participated (43% male and 57% female). Their ages ranged from 18–19 years with an approximate mean age of 18.5 years. Approximately 28% of the respondents reported that they exercised regularly.

Results of the present study show that both males 30 (50%) and females 40 (50%) never sought information from their teachers about healthy foods. Specifically, only two (3.3%) of the male students and one (1.3%) of the female students reported that they consulted their teachers about healthy foods. Interestingly, only 5 (6.3%) of the female and 4 (6.7%) of the male participants indicated that they found out about healthy eating from nurses. Surprisingly, 22 (36.7%) of the male and 18 (22.5%) of the female participants stated that they never studied nutrition in school. Results indicated that approximately 30 (37.5%) of the female students sometimes discussed healthy foods with their friends, while only 11 (18.3%) of the male students indicated that they did so. Some of the female participants, that is, 16 (20%) of them stated that they assisted their family in shopping for food, while only eight (13.3%) of the male participants related that they did so.

About the transitional operations, 17 (28.3%) of the male students stated that they plan their meals most of the time and 13 (16.3%) females indicated that they plan their meals some of the time. Only five (8.3%) of the males and one (1.3%) female indicated that they never choose to drink soda instead of water. About preference of fruit juice versus soda, 23 (28.38%) of the females indicated that they choose to drink soda instead of fruit juice and only 12 (20%) of the males did so. In all, 16 (26.7%) of the male students indicated that they always choose to eat sweets instead of fruit while only four (5%) of the females did.

Concerning to productive operations, 22 (36.7%) of the male students stated that they always think that they are gaining too much weight if they ate fewer sweets, while only seven (8.8%) of the female students reported this tendency. Overall, 38 (63.1%) of the males and 49 (61.3%) of the females indicated that they always make sure that they are drinking clean water. In all, 18 (30%) of the males and 5 (6.3%) of the females reported that they ate foods rich in vitamin C. When we asked about eating breakfast every day, 21 (35%) of the males and 24 (30%) of the females reported that they had breakfast every day. In all, only 6 (7.5%) of the females and 10 (16.7%) of the males indicated that they drank eight glasses of water every day. Finally, only 3 (5%) of the males and 3 (5%) of the females reported that they ate green vegetables twice a day.

About one third, that is, 15 (38.5%) of the students who exercised regularly always had breakfast, while 17 (43.6%) of those who thought they were gaining too much weight always exercised regularly. Of those who exercised regularly, only 12 (30.8%) always ate foods rich in vitamin C.

Only four (10.3%) of the students who indicated that they exercised regularly related that they always learned healthy eating habits from nurses, while 44 (43.6%) related that they never asked nurses about healthy eating habits.

Of the students who indicated that they choose to drink soda, only 7 (17.9%) of them exercised regularly and only 11 (28.2%) of the students who indicated a tendency to drink soda instead of fruit juice related that they exercised regularly. About preference of fruit versus sweets, only 10 (25.6%) who related that they choose to eat sweets instead of fruits indicated that they exercised regularly.

The results of a bivariate *t* test revealed no significant differences between males and females towards the total nutrition score (maximum score of 100; males mean score was 50.60 and the mean for females was 47.21 [*p* = .08]). There was a significant difference between those who indicated that they exercised regularly (mean 53.42) and those who related that they did not exercise regularly (mean 46.83; *p* = .002; Table 1).

**TABLE 1:** Gender and exercise status association with MIN‐SC score

Variables	MIN‐SC score					
	*N*	Mean	*SEM*	MD	95% confidence interval of the difference	*p*‐value
Male	60	50.6083	1.66	3.395	−50–7.30	.88^a^
Female	80	47.2125	1.17			
Do you [exercise] regularly?						
Yes	39	53.42	2.037	6.591	2.3–10.8	.002^a^
No	101	46.83	1.066			

^a^ Independent sample test (*t* test).

A chi‐square test showed a significant association between gender and exercising regularly. That is male students 33 (55%) were more active and did regular exercise compared with 6 females students (7.5%) with a *p* < .01 (Table 2). For every unit increase in exercising, there was a (−6.4) decrease in the score on the MIN‐SC scale. Further, for those who did not exercise, there was a 6.4 unit than those who work out (Table 3). There was a significant association such that those who exercised regularly had a poor nutritional score compared with those are not exercise (*β* = −6.411) *SE* 2.541, *p* = .03. However, the results of the simple regression model revealed that age and gender were not predictors of the nutritional score and no significant association was found with MIN‐SC score (*p* = .9; *p* = .8).

**TABLE 2:** Gender and exercise status association with MIN‐SC score

Variables	Do you [exercise] work out regularly?		*p*‐value
	Yes	No	
Male	33 (55%)	27 (45%)	<.001
Female	6 (7.5%)	74 (92.5%)	

Note

Chi‐square.

**TABLE 3:** Regression analysis shows gender, age and exercise status association with MIN‐SC score

Variables	MIN‐SC score				*p*‐value
	*Β*	*SE*	Beta	*t*	
Constant	59.624	7.238	—	8.238	.000
Gender	−0.601	3.284	−0.026	−0.183	.855
Age	0.064	0.645	0.014	0.100	.921
Do you [exercise] work out regularly?	−6.411	2.541	−0.248	−2.524	.013*

Note

* Simple linear regression was used.

## 4 DISCUSSION

The present study was conducted to evaluate the nutritional habits among nursing freshman students at a Saudi university, results revealed that most of the students were fairly aware of their nutritional habits and only a few lacked knowledge. These results were more positive than those found in a previously conducted study among primary school children's in SA, which found that one‐third of the respondents suffered from malnutrition (Khayri et al., 2016). Since malnutrition may be due to lack of awareness and insufficient consumption of daily requirements, awareness about nutrition is essential for adolescents.

This study revealed that approximately 32% of the students were in the habit of always eating breakfast. These findings are inconsistent with similar study conducted in the northwestern region of SA reported that approximately 15.7% of the participants skip breakfast or never eat breakfast daily (El‐Qudah, Al‐Omran, Abu‐Alsoud, & Yousef, 2012). Similarly, studies from other countries report a low intake of breakfast among adolescents (Čakar et al., 2018; Chourdakis, Tzellos, Papazisis, Toulis, & Kouvelas, 2010; Monneuse, Bellisle & Koppert, 1997). Literature has suggested that breakfast is the most important morning meal of the day because it promotes a healthy diet by decreasing the dietary fat intake and decreasing snaking (Calderon, Catherine, & Jambazian, 2004; Schlundt, Hill, Sbrocco, Pope‐Cordle, & Sharp, 1992).

The results also found that very few students prefer to eat chips and other snacks instead of fruits. These results were inconsistent with other previous studies conducted in SA, which found that students often depend on fast food, chips and other snacks (Khayri et al., 2016). Most studies in different countries, including SA, reported that changes in food and nutritional patterns can contribute to an increase in obesity which, in turn, may lead to the development of chronic diseases (Madani, 2000; Park, Yun, Park, Kim, & Choi, 2003). In addition to this, finding also reveals that most of the students think before they eat and consider if what they eat is healthy and majorities care about their intake of protein in meals. These results also indicate that most of the students eat foods rich in vitamin A, C and iron, thereby suggesting that many SA students practise healthy eating habits. However, a previous study conducted in SA found conflicting results and they found that lifestyle is a major influencer (Khayri et al., 2016). Our findings suggest that regular physical activity predicts the respondent's nutritional score.

Fruits and vegetables are rich sources of fibre and water and their consumption can reduce energy intake and prevent obesity (Rolls, Ello‐Martin, & Tohill, 2004). In our study, only 5% of male and female students reported that they consumed vegetables once or more than once a day, while a study in another country (Bin, Musaiger & D'Souza, 2009) reported that 35% of the students consumed vegetables every day. They also found gender differences in consumption with males consuming lesser fruits and vegetables; similarly, our study found that females showed greater interest in consuming soda instead of fruit juice. The finding of this study reveals no significant differences between males and females on the MIN‐SC score. There was a significant difference between those who exercised and those who did not. Further, chi‐square results revealed a significant association between gender and regularity of exercise. Results of the simple regression revealed that age and gender are not predictors of the nutritional score. The results suggest that nutritional habits among secondary school students in SA were reasonably good irrespective of age and gender. Conversely, research from the last two decades indicates that SA has seen rapid development in all aspects of life, which has resulted in the adoption of a sedentary lifestyle and unhealthy eating habits (e.g. intake of a high‐fat and low‐fibre diet), which in turn, is believed to be associated with malnutrition‐related health problems (Boutelle, Birnbaum, Lytle, Murray, & Story, 2003). Therefore, maintaining an optimum nutritional habit among students is vital, further earlier reports indicated the importance of healthy eating habits, which in turn helps in promoting quality of life for both children and adults (Bashatah & Alahmary, 2020; Lakshman, Sharp, Ong, & Forouhi, 2010; Lobstein, Baur, & Uauy, 2004). In addition to this, cheering healthy dietary habits among growing students may put off from number of disease (Bashatah & Alahmary, 2020). Evidence has also found that academia is one of the most common accessible settings for promoting health eating habits among students (Bashatah & Alahmary, 2020; Lakshman et al., 2010; Lobstein et al., 2004).

### 4.1 Limitations

The study had some limitations firstly; it was a cross‐sectional in design in a single institution in, Saudi Arabia. Additionally, the response rate to this study was very low limited to only first‐year nursing students. The generalization of the results to other health discipline in other areas of Saudi Arabia is unclear and more studies should be conducted in larger and more students with different disciplines are needed and are recommended to carry future studies with the help Arabic version of questionnaire, which designed to improve eating habits among Arabic‐speaking communities.

## 5 CONCLUSION

The findings serve as an indication of the need for nutrition education among nursing students. Students face enormous challenges to perform at maximum levels in their academics and other activities. However, to reach optimal performance, students must practise excellent nutritional habits. The present study found that Saudi nursing students had reasonably good nutritional consumption habits. Nonetheless, we suggest continued improvement in educating students about healthy diets and lifestyles. Despite the good consumption habits, it may be helpful to establish nutrition educational intervention programs in schools and colleges to build on this existing knowledge and to improve their habits.

## CONFLICT OF INTEREST

There are no potential conflicts of interest concerning the research, authorship and/or publication of this article.

## ETHICAL APPROVAL

The study was approved by the IRB committee of King Saudi university college of medicine Riyadh, Saudi Arabia (Ref no.: IRB E‐19‐3979) and was conducted in accordance with the ethical standards given in 1964 Declaration of Helsinki, as revised in 2008. Respondents were informed that response to the questionnaire would be considered as consent for participating in this study.

## Supporting information

Appendix S1

## Data Availability

The data used to support the findings of this study are available from the corresponding author on reasonable request.
