# Supplementary material for: Nutritional habits among nursing students using Moore Index for Nutrition Self Care: A cross‐sectional study from the nursing school Riyadh, Saudi Arabia
Source: Nurs Open. 2020 Jul 26;7(6):1846–51. doi: 10.1002/nop2.572 (PMC7544876; doi:10.1002/nop2.572)
Supplement: Supplementary file 1 — Appendix S1 [file NOP2-7-1846-s001.docx]

| Items | never | rarely | sometimes | most of the time | always |  |
| --- | --- | --- | --- | --- | --- | --- |
| I plan my meals so that they are healthy |  |  |  |  |  |  |
| I read about nutrition in books |  |  |  |  |  |  |
| I choose to drink soda instead of water |  |  |  |  |  |  |
| I study food labels to learn about nutrients in food |  |  |  |  |  |  |
| I learn about healthy food from watching TV |  |  |  |  |  |  |
| I suggest healthy foods for my family to buy |  |  |  |  |  |  |
| I eat foods that I know are good for me even if I do not like them |  |  |  |  |  |  |
| I try new foods |  |  |  |  |  |  |
| I ask my teacher about healthy food to eat |  |  |  |  |  |  |
| I eat foods containing iron |  |  |  |  |  |  |
| I choose to eat foods that contain vitamins |  |  |  |  |  |  |
| If I think I’m gaining too much weight I eat fewer sweets |  |  |  |  |  |  |
| I ask my grandparents questions about healthy eating |  |  |  |  |  |  |
| When I buy a snack I choose a soda rather than fruit |  |  |  |  |  |  |
| I put a lot of salt on the food that I eat |  |  |  |  |  |  |
| I eat the same foods every day |  |  |  |  |  |  |
| I find out about healthy eating from nurses |  |  |  |  |  |  |
| I make sure the water I drink is clean |  |  |  |  |  |  |
| I study nutrition in school |  |  |  |  |  |  |
| I ask my mother which foods are healthy |  |  |  |  |  |  |
| I eat foods that are good sources of vitamin C |  |  |  |  |  |  |
| I wash fruit before eating it |  |  |  |  |  |  |
| I make sure that meat I eat is cooked enough |  |  |  |  |  |  |
| I talk to my friends about which healthy foods to eat |  |  |  |  |  |  |
| I eat protein at every meal |  |  |  |  |  |  |
| I try to eat food and drink beverages with calcium |  |  |  |  |  |  |
| I eat foods that are good sources of vitamin A |  |  |  |  |  |  |
| I consider whether my meals have enough protein |  |  |  |  |  |  |
| I eat breakfast every day |  |  |  |  |  |  |
| I drink soda instead of fruit juices |  |  |  |  |  |  |
| I would choose to eat sweets instead of a piece of fruit |  |  |  |  |  |  |
| I think about whether what I eat is healthy |  |  |  |  |  | |
| I drink coffee with meals |  |  |  |  |  | |
| I choose to eat foods that are low in fats |  |  |  |  |  | |
| I obtain information about nutrition from the Internet |  |  |  |  |  | |
| I read public announcements about nutritious foods |  |  |  |  |  | |
| I eat a variety of foods |  |  |  |  |  | |
| I drink eight glasses of liquid every day |  |  |  |  |  | |
| I choose to eat chips and other snacks instead of fruit |  |  |  |  |  | |
| I read about nutritious food to eat in magazines or newspapers |  |  |  |  |  | |
| I help my family select food to buy |  |  |  |  |  | |
| I ask other adults questions about healthy eating |  |  |  |  |  | |
| I eat fruit |  |  |  |  |  | |
| I eat green vegetables |  |  |  |  |  | |
| I eat other vegetables |  |  |  |  |  | |
| I eat meat |  |  |  |  |  | |
| I drink milk |  |  |  |  |  | |
| I eat sweets |  |  |  |  |  | |
| I eat cereal, bread, or tortillas |  |  |  |  |  | |
| I eat high-calorie snack foods |  |  |  |  |  | |
